# Targeted Nanoparticles for the Binding of Injured Vascular Endothelium after Percutaneous Coronary Intervention

**DOI:** 10.3390/molecules27238144

**Published:** 2022-11-23

**Authors:** Pennapa Mungchan, Kittirat Glab-ampai, Nuttapol Chruewkamlow, Kongtana Trakarnsanga, Chatchawan Srisawat, Kytai T. Nguyen, Wanpen Chaicumpa, Primana Punnakitikashem

**Affiliations:** 1 Department of Biochemistry, Faculty of Medicine Siriraj Hospital, Mahidol University, Bangkok 10700, Thailand; 2 Department of Parasitology, Faculty of Medicine Siriraj Hospital, Mahidol University, Bangkok 10700, Thailand; 3 Research Department, Faculty of Medicine Siriraj Hospital, Mahidol University, Bangkok 10700, Thailand; 4 Siriraj Center of Research Excellence in Theranostic Nanomedicine, Faculty of Medicine Siriraj Hospital, Mahidol University, Bangkok 10700, Thailand; 5 Department of Bioengineering, University of Texas at Arlington, Arlington, TX 76019, USA

**Keywords:** percutaneous coronary intervention, vascular injury, biodegradable nanoparticles, endothelium regeneration, single-chain antibody variable fragment

## Abstract

Percutaneous coronary intervention (PCI) is a common procedure for the management of coronary artery obstruction. However, it usually causes vascular wall injury leading to restenosis that limits the long-term success of the PCI endeavor. The ultimate objective of this study was to develop the targeting nanoparticles (NPs) that were destined for the injured subendothelium and attract endothelial progenitor cells (EPCs) to the damaged location for endothelium regeneration. Biodegradable poly(lactic-co-glycolic acid) (PLGA) NPs were conjugated with double targeting moieties, which are glycoprotein Ib alpha chain (GPIbα) and human single-chain antibody variable fragment (HuscFv) specific to the cluster of differentiation 34 (CD34). GPIb is a platelet receptor that interacts with the von Willebrand factor (vWF), highly deposited on the damaged subendothelial surface, while CD34 is a surface marker of EPCs. A candidate anti-CD34 HuscFv was successfully constructed using a phage display biopanning technique. The HuscFv could be purified and showed binding affinity to the CD34-positive cells. The GPIb-conjugated NPs (GPIb-NPs) could target vWF and prevent platelet adherence to vWF in vitro. Furthermore, the HuscFv-conjugated NPs (HuscFv-NPs) could capture CD34-positive cells. The bispecific NPs have high potential to locate at the damaged subendothelial surface and capture EPCs for accelerating the vessel repair.

## 1. Introduction

Coronary artery disease (CAD) is one of the most common types of the cardiovascular diseases (CVDs), the leading cause of death globally [1,2]. According to the World Health Organization (WHO), there was an estimated 17.9 million deaths due to CVDs in 2019, which represents about 32% of all deaths worldwide [3]. Currently, percutaneous coronary intervention (PCI) is an effective treatment measure for CAD. Percutaneous coronary intervention, involving balloon angioplasty and stenting, is commonly used to manage the vascular obstruction. It is a minimally invasive surgical procedure, i.e., using a catheter to place a stent to open up the narrowed blood vessel [4,5]. However, post-treatment with PCI is frequently followed by vascular wall injury leading to inflammation, thrombosis, and finally restenosis (re-narrowing of the treated blood vessel) [6,7]. Thus, restenosis after PCI is the main problem that limits the long-term success of the PCI.

Percutaneous coronary intervention often causes vascular wall injury that induces thrombosis, starting with the stimulation of prothrombogenic reactions at the damaged vascular endothelium. P-selectin is upregulated and highly expressed on the surface of the activated endothelial cells. Moreover, the von Willebrand factor (vWF) is highly deposited on the subendothelial surface at the vascular damaged site [8]. This can trigger platelet adhesion at the injured location via interactions between adhesion receptors on the platelet surface and adhesive molecules on activated endothelial cells or extracellular matrix (ECM) components. The platelet glycoprotein (GP) Ib-V-IX receptor complex binds with immobilized vWF on the subendothelium, which slows down platelets under high shear stress. This allows the stable arrest of platelets, platelet activation, and irreversible platelet aggregation leading to thrombus growth [9]. Growth factors released from activated platelets promote vascular smooth muscle cell (SMC) proliferation and migration from the media to the intima, causing a thickening of the intimal vessel wall layer, called intimal hyperplasia, which restricts the luminal blood flow causing instent restenosis [6,7,10,11,12,13]. Thus, binding the platelet GPIb-V-IX complex to vWF is crucial for early platelet adherence to the damaged vessel wall. Because the GPIb alpha chain (GPIbα) subunit of the GPIb-V-IX complex is the binding site for vWF, it would be a potential targeting ligand that can localize at the vascular damaged site [14,15].

Nowadays, several approaches have been investigated to reduce the restenosis problem after PCI. Drug-eluting stents (DES) have been developed by coating drugs onto bare-metal stents to release antimitotic agents at local vascular tissues and decrease the restenosis rate [8,16,17,18,19]. However, a major limitation of the DES is that the limited amounts of released drugs can cause late stent thrombosis, which is potentially related to delayed endothelial regeneration, thus impairing vascular healing [8,20,21,22,23]. Later, endothelial progenitor cells (EPCs)-capturing grafts or stents have been produced to locally recruit EPCs to the damaged vascular site by coating EPC-specific ligands such as antibodies specific to the cluster of differentiation 34 (CD34) and vascular endothelial-cadherin (VE-cadherin) on the stents, which can enhance vascular repair and reduce intimal hyperplasia [24,25]. However, the murine monoclonal antibody (mAb) that was utilized is immunogenic in human [26]. Thus, the clinical efficacy and safety of the EPC capturing molecules is still needed to be improved.

Production of humanized and fully human antibodies has been evolved tremendously for medical purposes to reduce the immunogenicity of the non-human isotype [27]. Moreover, the intact four chain-mAb molecule has demonstrated clinical disadvantages, such as poor tissue penetration and slow blood clearance due to the large size (~150 kDa), and particularly induced proinflammatory responses due to the presence of the Fc portion, which can interact and activate effector cells and the complement cascade [27,28]. Thus, Ab engineering has been investigated to reduce the immunogenicity of Ab for medical use. A single-chain antibody variable fragment (scFv) is a small sized Ab (25–35 kDa) with one antigen-binding site that consists of variable domains of heavy and light chains of immunoglobulins (Ig), i.e., VH and VL domains, respectively, that are linked together by a short linker peptide [(Gly_4_Ser)_3_] [29]. The scFv retains the antigen-binding capability and specificity of the original Ab counterpart. Human scFv (HuscFv) has demonstrated several advantages in therapeutic application, such as more rapid tissue penetration and blood clearance, and negligible immunogenicity, if there were any, in human recipient and does not induce inflammation due to the absence of the Fc portion [28,29]. Thus, in this study, fully human scFv (HuscFv) specific to CD34, a surface marker of EPCs, was used in the preparation of the NPs that captured the EPCs [8,25,30,31].

Recently, nanoparticles (NPs) have been widely used as a delivery vehicle of drugs or other therapeutic agents. The polymeric NPs prepared from poly(lactic-co-glycolic acid) (PLGA) is of particular interest due to its approval by the US Food and Drug Administration (FDA) in the biocompatibility and biodegradability for medical applications [32]. Nanoparticles possess many beneficial properties, including a small size in the nanometer range, sustained release of encapsulated drugs/reagent, and sometimes facilitating intracellular delivery [33]. Furthermore, the surface of NPs could be modified for desired properties. For example, conjugation of targeting moieties onto NPs, such as proteins, peptides, or aptamers, is applied for the targeted delivery toward a specific target [34].

The goal of this study was to develop the new platelet-mimicking and EPC-capturing NPs that can target injured blood vessels as well as stimulate endothelium regeneration. Therefore, the biodegradable polymeric PLGA NPs were designed to be coated with two ligands. The first ligand is GPIbα, the binding site of a platelet receptor complex to vWF that is highly deposited on the damaged subendothelial surface. The other ligand is the HuscFv specific to CD34, a surface marker of EPCs. It was hypothesized that the dual-conjugated NPs (dual-NPs) should be able to target the injured vascular site and capture and recruit EPC simultaneously for accelerating endothelium regeneration. This development of targeting NPs may provide a strategy for treating the PCI-injured vascular endothelium.

## 2. Results

### 2.1. Production of Anti-CD34 HuscFv

**Figure 1:**
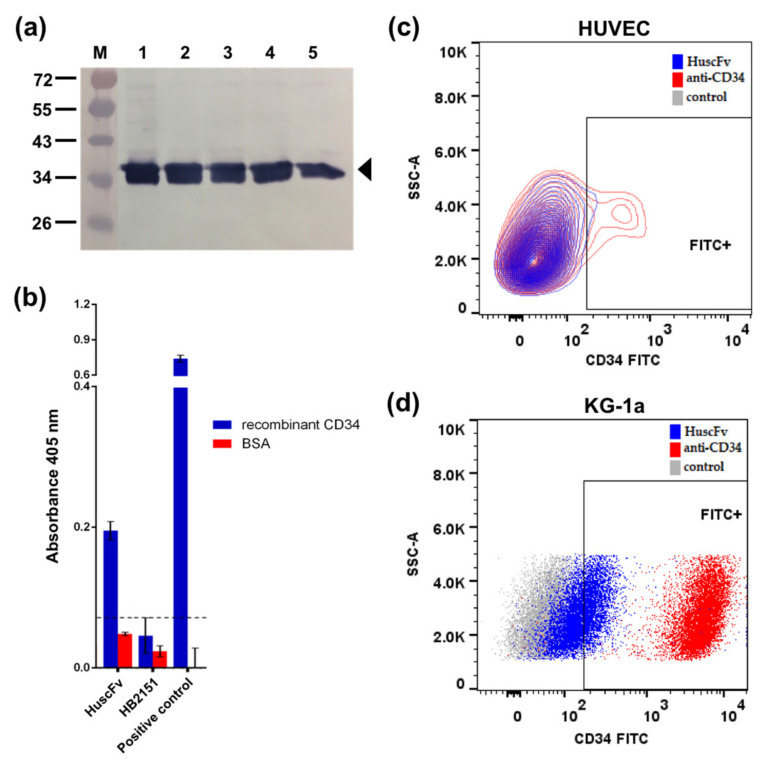
Characterization and binding tests of soluble anti-CD34 HuscFv. (**a**) The HuscFv (arrowhead) produced by the *huscfv*-positive Rosetta *E. coli* clone as determined by Western blotting. Lane M, Pre-stained standard protein marker; Lanes 1–5, HuscFv of randomly picked transformed *E. coli* colonies. (**b**) Binding test of crude HuscFv from selected phage-transformed HB2151 *E. coli* clone to recombinant CD34 by indirect ELISA. BSA served as negative control antigen. HB2151, lysate of original HB2151 *E*. *coli* as background binding control; Positive control, mouse anti-CD34 Ab; Dotted blackline, the cut-off absorbance between positive and negative ELISA that determined from the highest absorbance of HB2151 *E. coli* control. Presented data are mean ± SD (*n* = 3). Representative of binding results of purified HuscFv to (**c**) HUVECs and (**d**) KG-1a cells by flow cytometry analysis.

The selection of HuscFv displaying phage clones with binding affinity to CD34 from the HuscFv phage display library was performed successfully by phage bio-panning against recombinant CD34 (rCD34). The selected *huscfv* positive-*E. coli* clone was able to express soluble HuscFv with an estimated molecular size 34 kDa, as determined by Western blotting (Figure 1a). In addition, this candidate HuscFv clone was subsequently purified and showed high purity.

### 2.2. Binding of the Anti-CD34 HuscFv to rCD34 and CD34-Positive Cells

Crude HuscFv of selected *E. coli* clone demonstrated high binding to rCD34 while low binding to bovine serum albumin, BSA, (Figure 1b), suggesting that the HuscFv might bind specifically to CD34-positive cells. Cell binding tests revealed that the HuscFv could bind to both human umbilical vein endothelial cells (HUVECs) and acute myeloblastic leukemia cells (KG-1a), as shown by higher numbers of FITC-positive cells when treated with the HuscFv, compared to the control PBS group (Figure 1c,d).

### 2.3. Physical Characterization, EE and CE of Conjugated NPs

The PLGA NPs could be synthesized successfully by a single/double emulsion solvent evaporation technique with an average size of 189–199 nm and the standard deviation of nanoparticle sizes around 49.0–52.5 nm. The average zeta potential, or surface charge, was in the range of −39 to −31 mV with the standard deviation of 7.1–8.0 mV, as shown in Table 1. The fabricated NPs demonstrated a low polydispersity index and high negative surface charge, indicating narrow NP size distribution and stability in dispersion. The encapsulation efficiency of NPs was performed and displayed 89.3 ± 0.3% of total coumarin-6 (C6) loaded into NPs and 48.8 ± 0.3% of total rhodamine B (RhB) loaded into NPs. Herein, C6-loaded NPs and RhB-loaded NPs were used for NPs tracking in the binding tests.

**Figure 2:**
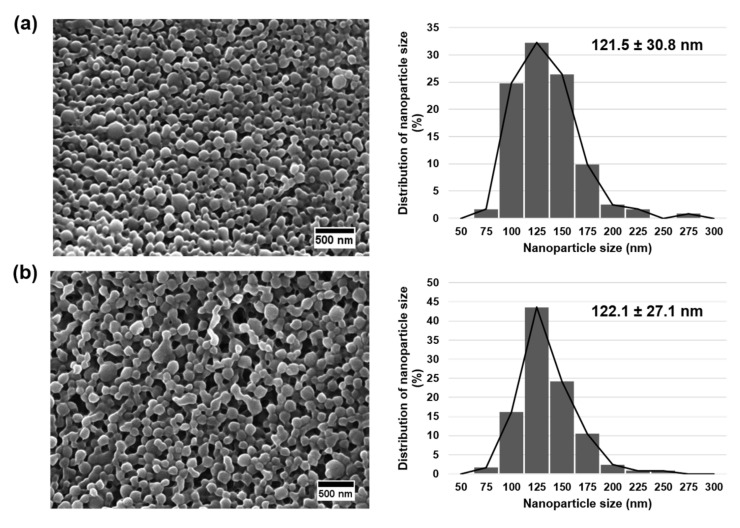
Morphology and distribution of NPs via FE-SEM. (**a**) Control NPs and (**b**) conjugated NPs examined by FE-SEM. Scale bar represents 500 nm.

The GPIbα and HuscFv molecules could be successfully conjugated onto the PLGA NPs by carbodiimide reaction. The average size of GPIbα, HuscFv and dual targeting ligands conjugated on the surface of NPs was 200.2 ± 50.6, 202.3 ± 52.0 and 216.6 ± 64.0 nm, respectively. The average zeta potentials of those NPs were −40.8 ± 7.8, −41.6 ± 6.9 and −38.9 ± 7.8 mV, respectively, as shown in Table 1. The conjugation efficiency of GPIbα, HuscFv and both targeting moieties on the surface of NPs were 40.1 ± 24.3, 38.2 ± 24.5 and 61.3 ± 20.2%, respectively, which also shown in Table 1. In addition, the morphology of fabricated NPs presented as spherical shapes with a smooth surface examined by the Field Emission Scanning Electron Microscopes (FE-SEM), as shown in Figure 2. The distribution of control and conjugated NPs were examined from the FE-SEM images and revealed the average sizes of 121.5 ± 30.8 and 122.1 ± 27.1 nm, respectively.

### 2.4. Biocompatibility of Conjugated NPs

#### 2.4.1. Blood Clotting Test

**Figure 3:**
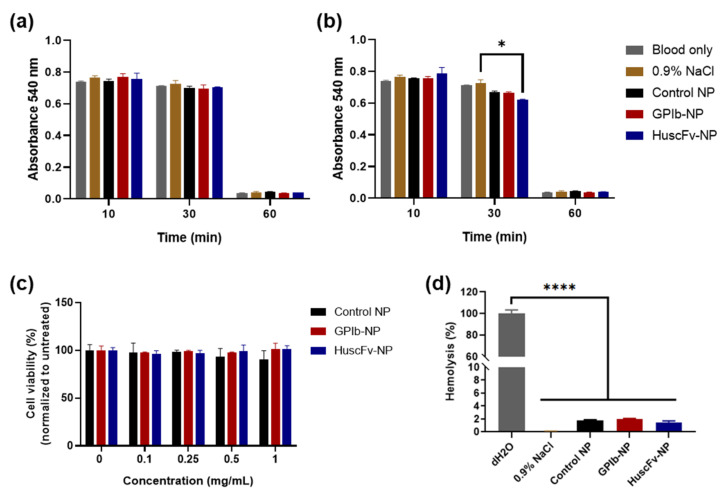
Cytocompatibility and hemocompatibility of NPs. Absorbance 540 nm of whole blood samples after treatment with PLGA NP samples for 10, 30 and 60 min at the NP concentrations of (**a**) 0.5 mg/mL and (**b**) 1.0 mg/mL. Normal saline (0.9% NaCl) served as negative control. (**c**) Cytotoxicity of HUVECs exposed to various concentrations of NP preparations for 24 h by MTS assay. (**d**) Hemolysis test of whole blood samples treated with various NP preparations. The absorbance of 545 nm for determining hemolysis when treated anticoagulated blood with NP preparations for 2 h at the NP concentration of 1.0 mg/mL. The anticoagulated blood treated with 0.9% NaCl and dH_2_O served as negative and positive hemolysis control, respectively. Blank, uncoated NPs served as control NPs. Data are presented as mean ± SD (*n* = 3). * *p* < 0.05 and **** *p* < 0.0001.

Blood clotting has occurred along with the time duration after clotting initiation. The nonclotted red blood cells were lysed and measured for the released hemoglobin at an absorbance 540 nm. The absorbance kept decreasing until 60 min after clotting initiation. After 30 min, the blood started clotting and clotted completely at 60 min. There was no significant difference in blood clotting between NP-treated and control groups from the absorbance at each time point (Figure 3a,b). However, the HuscFv-NPs showed significantly decreased absorbance, reflecting higher blood clotting than the 0.9% saline group at the 30-min time point. Overall, the result indicated that PLGA NPs did not cause blood clotting at 0.5 mg/mL concentration.

#### 2.4.2. Hemolysis Test

There were no lysed red blood cells in the supernatant, and similarly in NP-treated blood and negative control. For distilled H_2_O positive control, there were lysed red blood cells in the supernatant, calculated as 100% hemolysis. The percentage of hemolysis of blood treated with all NP samples was lower than 1.98%, which was significantly different compared to the positive control, with no significant difference to the 0.9% saline negative control. The hemolysis for GPIb-NPs was the highest (1.98%), whereas the other NP groups had lower hemolysis percentages (less than 1.78%) (Figure 3d), implying that PLGA NPs did not cause hemolysis.

#### 2.4.3. Cytocompatibility

The cytocompatibility of conjugated NPs was evaluated by cytotoxicity test with HUVECs. All NP groups revealed no significant difference of cell viability at the NP concentration of 0.1, 0.25, 0.5, 1.0 mg/mL compared with the untreated control (Figure 3c), indicating that PLGA NPs were not toxic to HUVECs.

### 2.5. Binding of HuscFv-NPs to CD34-Positive Cells

**Figure 4:**
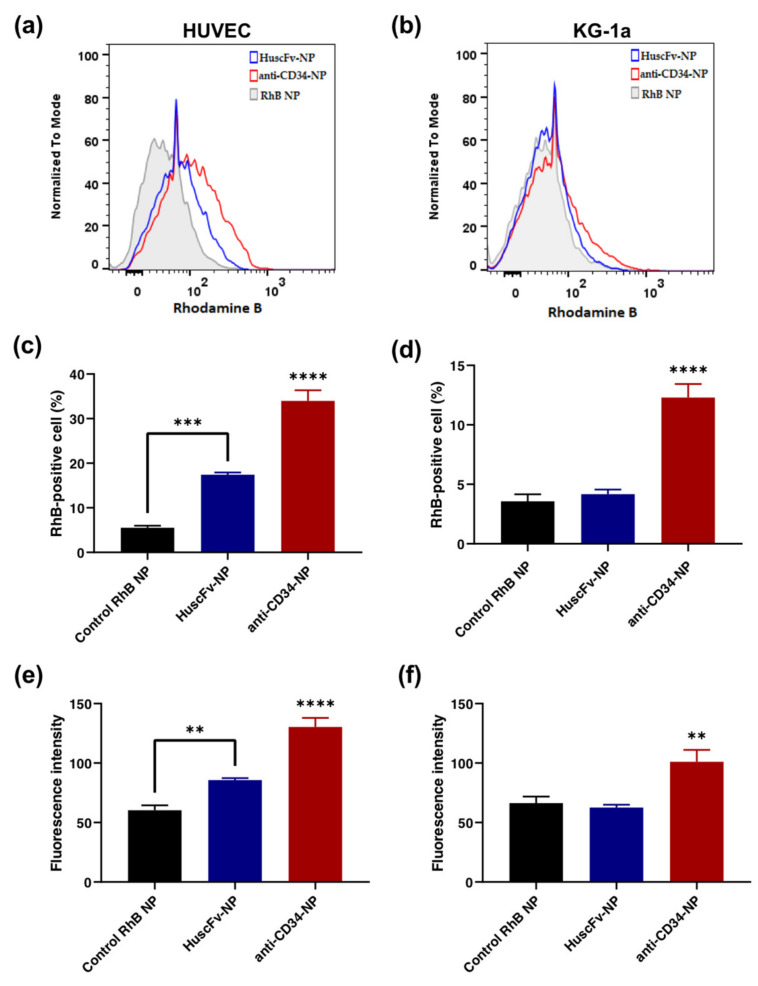
Binding tests of HuscFv-NPs to (**a**) HUVECs and (**b**) KG-1a cells by flow cytometry. The percentage of RhB-positive cells of (**c**) HUVECs and (**d**) KG-1a cells, and mean fluorescence intensity (MFI) of conjugated-NPs binding to (**e**) HUVECs and (**f**) KG-1a cells. Positive and negative controls were anti-CD34 Ab-conjugated NPs (anti-CD34-NP) and RhB-loaded NPs (RhB NP), respectively. Data are presented as mean ± SD (*n* = 3). ** *p* < 0.01, *** *p* < 0.001 and **** *p* < 0.0001.

The RhB-loaded NPs conjugated with HuscFv were tested for binding with HUVECs and KG-1a cells by detecting RhB fluorescence with flow cytometry. The result demonstrated that there were significantly higher RhB-positive cells in the HuscFv-NP group compared to the control NP group, at 11.88% in HUVECs (Figure 4a,c), with significantly higher mean fluorescence intensity (MFI) (Figure 4e). Furthermore, in KG-1a cells, the HuscFv-NP group tended to have more RhB-positive cells (~0.6%) than the control NP group (Figure 4b,d). However, there was no significant difference in the MFI (Figure 4f).

### 2.6. Binding of GPIb-NPs with vWF

**Figure 5:**
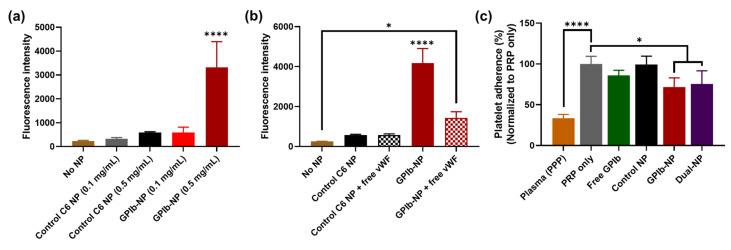
In vitro targeting efficiency of GPIb-NPs to vWF and prevention of platelet adherence to vWF-coated surface. (**a**) Fluorescence intensity (a.u.) of GPIb-NPs adhering on vWF-coated surfaces. Data are presented as mean ± SD (*n* = 6). (**b**) Binding competition of GPIb-NPs to vWF-coated surface in the presence of free vWF as determined by fluorescence intensity (a.u.). C6-loaded NPs (C6 NP) served as control NPs. (**c**) The percentage of platelet adherence to vWF-coated surface in the presence of different NP samples quantified by LDH assays, which was determined at absorbance at 490 nm, then normalized to the PRP-only group. Platelet-poor-plasma (PPP) and PRP only served as negative and positive controls, respectively. Data are presented as mean ± SD (*n* = 3). * *p* < 0.05 and **** *p* < 0.0001.

The binding of NPs to the vWF-coated surface was measured directly from the fluorescence intensity of the loaded C6 inside NPs. The result demonstrated that GPIb-NPs revealed more fluorescence intensity than control NPs (at the concentration of 0.5 mg/mL) (Figure 5a), implying that the GPIb-NPs had a higher binding ability to vWF. In the GPIb-NP group with free vWF, the fluorescence intensity was decreased significantly compared to the GPIb-NP group without vWF. On the other hand, there was no difference in the fluorescence intensity with or without free vWF in the control group (Figure 5b), indicating that GPIb-NPs could bind to free vWF, resulting in lower binding to coated-vWF. These results suggested that GPIb-NPs had a high affinity to vWF and thus should be able to target the vWF deposited on the damaged endothelial site.

### 2.7. GPIb-NPs Prevention Platelet Adherence to vWF Measure

In this experiment, dual-NPs were determined for the functional activity in reducing platelet adherence to vWF compared to GPIb-NP. The platelets bound to the vWF-coated surface was quantified by the lactate dehydrogenase (LDH) assay. The platelet-rich-plasma (PRP) only group was used as a positive control, representing 100% platelet adherence. GPIb-NPs and dual-NPs could significantly reduce platelet adherence (decreased to an estimated 72% and 76%, respectively) compared to the PRP-only group. In addition, the presence of free GPIb could decrease bound platelets to 86%. Furthermore, there was no significant difference in platelet adherence in the control NP group compared to the PRP-only group (Figure 5c). The result indicated that GPIb could target vWF-coated surfaces, thus, inhibiting platelet adherence to vWF. Furthermore, dual-NPs could reduce platelet adherence, similar to GPIb-NP, which indicated that the NPs conjugated with dual moieties did not interfere with the specific binding of each ligand.

## 3. Discussion

Recently, scFv has been established and applied for medical therapeutics and diagnostics due to its several benefits over an intact Ab molecule. The lack of the Fc portion allows the mAb molecules to be significantly less immunogenic in humans [29,35,36]. Thus, the scFv molecules demonstrate the potential for imaging and cell targeting in clinical use [28,37]. Phage display technology allows the in vitro selection of high-affinity scFv from an extensive phage library by circumventing immunization in conventional techniques [29,38]. Several studies have developed novel fully human scFv (HuscFv) against different antigens by phage display technology [39,40,41]. This study demonstrates the construction of HuscFv specific to CD34 by phage display technology.

In the present study, a phage transformed *E. coli* clone with high HuscFv expression capability was selected. The HuscFv could be expressed by the *E. coli* clones in soluble form, as detected by Western blotting (Figure 1a). The unpurified HuscFv of a selected *E. coli* clone could bind to rCD34 and yielded significant indirect ELISA signal above the control antigen (BSA) (Figure 1b). The HuscFv was then purified with TALON^®^ (Clontech Laboratories, Mountain View, CA, USA) resin beads. The purified HuscFv revealed the binding capability to HUVECs and KG-1a cells compared to the negative control (Figure 1c,d). According to the result, the KG-1a cells showed a higher positive percentage of HuscFv binding, as these cells are the highly expressed CD34-positive cell line, whereas HUVECs are moderately expressed CD34-positive cells [42]. It was reported that the CD34 is expressed in a small subset of cultured HUVECs, which are endothelial tip cells located at the leading edge of the vascular sprout that can extend filopodia [43]. Only about 20% of the cultured HUVECs is CD34-positive [44]. In comparison to our binding result, Yu et al. developed the scFv specific for hepatocellular carcinoma, which reported an estimated 32% binding with HepG2 cells, compared to normal liver cells, tested by flow cytometry [45]. The preliminary binding results by flow cytometry suggested that this candidate HuscFv could bind to CD34-positive cells; thus, the HuscFv was further conjugated onto PLGA NPs. In this study, our construction of HuscFv against CD34 is a preliminary step to develop HuscFv that can target CD34 antigen as a new EPC-capturing molecule with negligible immunogenicity (the HuscFv is a fully human protein) for clinical application. Further investigation and (perhaps) engineering of the HuscFv may be essential for improving the binding affinity and specificity to CD34. Moreover, in order to promote endothelialization, the orientation and specificity of targeting ligand to the binding sites are also important factors [46]. Thus, NPs are corresponded to the controllable orientation of anchored moieties. Despite the benefit of NPs in terms of controllable fashion, using a non-suitable targeting ligand may hinder its efficiency. Likewise, using the full-length of the antibody could be cumbersome, as it may conjugate with the NPs in an uncontrollable fashion [47]. Moreover, applying HuscFv alone without the aid of a carrier tends to cause agglomeration [48]. Therefore, NPs served as a based-material would prevent aggregation that may occur.

PLGA NPs, approved by the US FDA, have been extensively developed and used as drug delivery vehicle and diagnostics in CVDs [47,48]. For instance, the delivery of NPs loaded with anti-proliferative agent after percutaneous transluminal coronary angioplasty or balloon angioplasty to treat restenosis [49,50,51]. Moreover, due to its ease of surface conjugation with the chemical reactions, the PLGA NPs are a suitable candidate for anchoring HuscFv with controlled conjugation orientation [52]. Besides, the colloidal stability of PLGA NPs is the essential factor. PLGA NPs demonstrated great stability in both in vitro and in vivo study [53]. Although lipid nanoparticles’ engineering with scFv for targeting endothelial cells was also studied [54], there are common drawbacks such as aggregation of using lipid nanoparticles as based-materials may provide unsatisfactory result. According the several advantages of PLGA NPs as aforementioned, we therefore decided to use it in this work. In this study, the PLGA NPs were successfully fabricated and conjugated with GPIb and HuscFv. Poly(vinyl alcohol) or PVA was used as a stabilizer, improving the water solubility. The fabricated unloaded PLGA contained a highly negative charge (of about −31.3 ± 7.2) due to the presence of carboxylic acid groups [55]. In combination with the stabilizer PVA, the synthesized highly negative charged PLGA is colloidally stable under aqueous solution. In addition, the PLGA conjugated with GPIb and HuscFv was also accomplished. From characterization with DLS, the NP size ranged from 189 to 217 nm, and the average zeta potential was in the range of −30 to −42 mV (Table 1). These results are consistent with other studies [47,56]. Moreover, the presence of the high negative charge of the conjugated particles may delay the protein corona formation due to the negatively repulsive interaction between the fabricated particles and the proteins, making the fabricated system applicable [57]. In our study, the encapsulated of fluorescent dyes such as C6 and RhB decreased the surface charge of NPs, which was corresponding to the study of Heng et al. that the C6-loaded polymeric NPs demonstrated a decreased zeta potential [58]. The conjugation of GPIb and HuscFv slightly increased the particle sizes (Table 1), which was reported in previous studies about the conjugation GPIb and anti-CD34 monoclonal antibody [8,47]. An increase in the particle size might represent the incorporation of ligand to the surface of NPs. Particles larger than 300 nm in diameter are often accumulated in the liver, spleen, and lung, making them unavailable at the arterial wall when delivered systemically [59]. In this research, the fabricated NPs were ~200 nm in diameter and should be suitable for accumulation at the injured vascular site. Our fabricated material is considered as submicron material with sizes ranging from 100 nm–1 µm [60]. However, according to several nanomaterial studies, they also reported polymeric NPs with sizes larger than 100 nm [49,52]. Therefore, the polymeric particles in the present study were represented as NPs. Furthermore, after ligand conjugation, the surface charge of NP was decreased, such as from an average of −29.7 to −38.9 mV in dual-NPs (Table 1), corresponding to the findings of Su et al. and Patel et al. that the particle surface charge decreased after conjugation with antibodies [8,61]. The decrease in surface charge might be due to the incorporation of the protein with a total negative charge onto the surface of NPs. However, SEM images of unconjugated and conjugated NPs demonstrated a similarity in morphology and size. Therefore, the presence of targeting ligands may not be suitable to be detected by using this technique. In addition, the dual conjugation of two ligands, GPIbα and HuscFv, onto the NP surface revealed increased conjugated double ligands that was approximately the summation of each single ligand conjugation. We assumed that both ligands could be conjugated equally (more or less) without competition. Further investigation to determine the conjugation efficiency of each ligand in double conjugation is also important.

The cytotoxicity result demonstrated negligible cytotoxicity of PLGA NPs in HUVECs. There was a similar cell viability of NP-treated cells compared with the untreated at NP concentrations of up to 1 mg/mL (Figure 3c). According to Kona et al., there was more than 90% cell viability of human aortic endothelial cells after exposure to PLGA NPs at concentrations of up to 0.5 mg/mL [47]. The previous studies demonstrated the hemocompatibility of polymeric NPs [62,63]. Similarly, our results of fabricated PLGA NPs did not cause blood clotting or hemolysis compared to the control group (Figure 3a,b,d). The blood clotting and hemolysis evaluation of fabricated NPs indicated that PLGA NPs were hemocompatible. Our results and previous studies suggested that PLGA NPs are hemocompatible and noncytotoxic, thereby potentially applicable clinically.

This study demonstrated the binding ability of HuscFv-NPs to CD34-positive cells as the model of EPCs. The fabricated HuscFv-NPs could bind to HUVECs and KG-1a cells, with higher MFI in HUVECs than negative control NPs (Figure 4). It was previously reported that the rate of EPC adhesion on the surface coated with GPIb/anti-CD34-conjugated NPs was nearly two times as fast as on control NPs within the first hour of incubation under constant agitation [8]. All results suggested the in vitro capturing ability of EPCs by NPs modified with CD34 targeting ligands, including anti-CD34 mAb and our novel anti-CD34 HuscFv.

From the binding test of GPIb-NPs with immobilized vWF in vitro, it was revealed that GPIb-NPs demonstrated more binding to vWF compared to unconjugated NPs (Figure 5a). In addition, the presence of free vWF could compete for binding with GPIb-NPs, thus fewer NPs bound to coated-vWF (Figure 5b). Our results are consistent with previous studies, reporting the interaction of polymeric NPs conjugated with GPIb to vWF in vitro, and also the adherence of the NPs to arteries with lumen injury ex vivo [8,47]. These results and our results suggested that GPIb-NPs had a high affinity to vWF.

The coverage of vWF surface by the nanoparticles can be observed in other work as well. The researchers studied the competitive deposition between nanoparticles and platelets. They found that nanoparticles had higher affinity compared to the platelets, demonstrating a smaller number of deposited platelets when the nanoparticles were applied [8]. Furthermore, it is noteworthy that the GPIb-NPs could significantly reduce platelet adhesion to vWF in vitro (estimated 72% platelet adherence) (Figure 5c). It was reported that GPIb/anti-CD34-conjugated NPs reduce platelet adherence to the vWF-coated surface compared to the control, and also fewer platelets on the vWF-coated surface confirmed by SEM [8]. It indicated that GPIb-NPs should be able to prevent platelet adhesion to vWF at the injured vascular site. In addition, to demonstrate the binding function of dual-NPs, these dual-NPs could reduce platelet adherence similar to GPIb-NP (Figure 5c). It indicated that dual conjugation on NPs did not interfere with the specific binding of each ligand. Corresponding to the study by Ito et al., in which the polymeric NPs were conjugated with both the FITC-labeled Ab and AP enzyme by incubating activated NPs in the solution mixture of two proteins, they found that individual proteins could independently react with their specific target molecule on the NPs. Furthermore, the single conjugation of the enzyme on NPs demonstrates almost the same level of enzymatic reaction as the dual-NPs [64].

To our knowledge, the conjugation of anti-CD34 HuscFv onto PLGA NPs has never been reported. However, several studies reported drug-loaded PLGA NPs modified with scFv targeting different antigens for cancer-targeted therapy [65,66]. For instance, Le et al. demonstrated that the docetaxel-loaded pegylated PLGA NPs conjugated with anti-HER2 scFv had significant internalization and cytotoxic effects on the spheroids of HER2-overexpressing cancer cells [66]. Therefore, using scFv to modify NP surface would be potential for the NP delivery to specific targets. It is also noted that the NPs were developed for coating on the surface of the stents and applied locally with the stent at the injured vascular site. Several stent coating methods, both physical and chemical methods, have been widely used, such as electrostatic dry powder deposition, layer-by-layer (LbL) assembly coating, electrospinning, anodic oxidation, silanization, etc. [67]. Coating GPIb-NPs on the stent surface with a suitable method accompanied by the favorable affinity with vWF at the injured site would enhance NPs’ localization and mitigate the effect from its neutralization by free vWF. Furthermore, the binding site for GPIbα in circulating plasma vWF is not active and non-accessible in normal conditions. During vascular injury, the immobilization of vWF to exposed subendothelial matrix and high shear forces cause a conformational activation of the vWF A1 domain, resulting in the binding with GPIbα [68]. Thus, our designed NPs will be deposited on the stent surface and should not be neutralized by plasma vWF. Therefore, the fabricated GPIb-NPs have the potential in developing NPs-coated stent by applying the NPs locally on the stent.

In future work, the oriented conjugation should be further studied to project the antigen-binding site of the HuscFv outward NP surface. Furthermore, the dual-NPs should be investigated for the binding ability to vWF and recruitment of EPCs simultaneously. In addition, the binding results with HUVECs and KG-1a cells might not fully represent the ability of constructed HuscFv to capture EPCs circulating in the blood. Thus, the HuscFv should be further evaluated for the binding affinity with isolated human EPCs directly. Moreover, the binding test in the flow condition should be conducted to demonstrate the capture ability to circulating cells.

The present fabrication of dual-NPs is the first step towards developing the vascular implant surfaces coated with the NPs to capture and recruit circulating EPCs to the endothelial damaged site to repair the vascular wall injury that occurred after the PCI. However, the major concern is whether the captured EPCs could function and typically differentiate to form reendothelialization [69,70]. Thus, the EPC function and behavior after capture and attachment onto implants coated with the NPs should also be studied in the future.

## 4. Materials and Methods

### 4.1. Cell Lines and Reagents

Human umbilical vein endothelial cells (HUVECs; ATCC^®^ CRL-1730^TM^ (Manassas, VA, USA)) were cultured in EndoGRO^TM^-VEGF complete medium (Merck Millipore, Burlington, MA, USA) supplemented with 1% penicillin-streptomycin (Invitrogen; Waltham, MA, USA). Acute myeloblastic leukemia cell line (KG-1a cells; ATCC^®^ CCL-246.1^TM^) were cultured in IMDM medium (ATCC, Manassas, VA, USA) supplemented with 20% fetal bovine serum (FBS) and 1% penicillin-streptomycin (Invitrogen; Waltham, MA, USA). Both cells were grown at 37 °C in a humidified 5% CO_2_ atmosphere. Poly (lactic-co-glycolic acid) (average MW: 24,000–38,000; lactic to glycolic ratio: 50:50), dichloromethane (DCM), and polyvinyl alcohol (PVA) were purchased from Sigma-Aldrich (St. Louis, MO, USA). Most materials and reagents were purchased from Sigma-Aldrich unless specified. The antibodies used in this study were purchased from Abcam (Cambridge, UK) unless specified.

### 4.2. Production of HuscFv That Bound to Recombinant CD34 (rCD34)

Human rCD34 expressing the extracellular domain (Sino Biological, Beijing, China) was used as antigens in phage biopanning to select HuscFv-displaying phages that bound to rCD34 from a HuscFv phage display library, constructed previously [71]. The biopanning selection was performed according to a previously described protocol [72]. The rCD34-bound phages from the panning were used to transfect F+ HB2151 *E. coli*, then the transformed bacteria were screened for the presence of the HuscFv-coding sequences (*huscfvs*) by direct colony PCR using the pCANTAB5E phagemid specific primers [71]. The *huscfvs* were subcloned into the pET-23b^+^ vector, then the recombinant vectors were transformed into Rosetta *E. coli*, which is a bacterial expression strain. The *huscfv*-positive *E. coli* clones were grown individually under the IPTG-induction condition for HuscFv expression by adding 1 mM IPTG and 1 mM PMSF. Then, the bacterial culture was incubated further at 16 °C with 220 rpm shaking for 72 h. Secreted soluble 6× His tagged-HuscFv in culture medium was detected via Western blotting by probing the sodium dodecyl sulfate polyacrylamide gel electrophoresis (SDS-PAGE)-separated components with anti-His-tag Ab (Bio-Rad, Hercules, CA, USA).

### 4.3. Binding Test of Crude HuscFv to rCD34

The unpurified soluble HuscFv from lysate of each HB2151 *E. coli* cell was tested for binding to rCD34 by using the indirect enzyme linked immunosorbent assay (indirect ELISA). Briefly, 1 µg of rCD34 was immobilized onto the surface of an ELISA plate (Thermo Scientific, Waltham, MA, USA). Bovine serum albumin (BSA; Merck Millipore, Burlington, MA, USA) served as negative control antigen. The unoccupied sites on the ELISA well surfaces were blocked with 5% skim milk, then the immobilized antigens were added with the *E. coli* lysates containing HuscFv and the plate was kept at 4 °C overnight. Anti-E-tag Ab-horseradish peroxidase conjugate was used as a secondary antibody, and ABTS [2,2′-Azinobis (3-ethylbenzothiazoline-6-sulfonic acid)-diammonium salt] substrate was used for color development. Whole-cell lysate of *E. coli* HB2151 served as the background binding control. The absorbance at 405 nm was measured by a microplate reader (BioTek, Winooski, VT, USA). The *E. coli* clones that their lysates containing HuscFv that gave ELISA signal at OD 405 nm to the rCD34 more than two times higher than the same lysate to control BSA was selected for further experiments.

### 4.4. Purification of Soluble HuscFv with His-Tag Affinity Resins

The rCD34-bound HuscFv in the lysate of the selected *E. coli* clone was purified by metal affinity chromatography using TALON^®^ His-tag affinity resin beads (Clontech Laboratories, Mountain View, CA, USA) according to the manufacturer’s instructions. The purity of HuscFvs was determined by SDS-PAGE followed by staining with Coomassie Brilliant Blue G-250 dye (Bio-Rad, Hercules, CA, USA).

### 4.5. Binding Test of Purified Soluble HuscFv to CD34-Positive Cells by Flow Cytometry

HUVECs (moderately expressed CD34-positive cells) and KG-1a cells (highly expressed CD34-positive cells) were used as the models of EPCs for the binding test with purified HuscFv [42,44]. Phosphate buffered saline, pH 7.4 (PBS) and anti-CD34 antibodies (R & D Systems, Minneapolis, MN, USA) served as the negative and positive binding controls, respectively. HUVECs were grown and detached with 5 mM EDTA for 15–30 min. The cells (1 × 10^5^ cells) were blocked with 20% FBS for 30 min. Grown KG-1a cells (1 × 10^5^ cells) were blocked with 1% BSA for 30 min. Ten µg of HuscFv in 100 μL FACS buffer (PBS with 2% FBS and 0.5 mM EDTA) were added to each cell preparation and kept at 4 °C for 2 h. After cell washing and blocking with 20% FBS between each step, anti-His tag Ab and secondary IgG-FITC conjugate were added, respectively, and the preparations were kept on ice for 1 h. Thereafter, stained cells were subjected to flow cytometric analysis (BD FACSCelesta™, BD Bioscience, San Jose, CA, USA) using FACSDiva software (BD Bioscience, San Jose, CA, USA).

### 4.6. Preparation of Drug-Loaded Polymeric NPs

Different NP preparations were prepared for different experiments. Unloaded NPs were tested for biocompatibility assessment, while fluorescent dye-loaded NPs, including rhodamine B (RhB)-loaded and coumarin-6 (C6)-loaded NPs, were prepared for NP tracking in the binding tests. The PLGA NPs were prepared by the single/double emulsion-solvent evaporation technique. Briefly, PLGA polymer was dissolved in 3% (*w*/*v*) dichloromethane (DCM). For the synthesis of unloaded and RhB-loaded NPs, distilled water (dH_2_O) and RhB solution were mixed with PLGA/DCM solution as emulsion by sonication. The emulsion was added drop-wised into 5% polyvinyl alcohol (PVA) solution on a stirrer, then sonicated. After that, the solvent was evaporated overnight. Finally, PLGA NPs were collected using a superspeed centrifuge (30,000× *g*) (Sorvall RC 6 Plus, Thermo Scientific, Waltham, MA, USA); then, the NPs were lyophilized and stored at −20 °C. To synthesize C6-loaded NPs by single emulsion-solvent evaporation method, C6 in DCM was mixed directly with PLGA/DCM solution without sonication, then added drop-wised into 5% PVA solution.

### 4.7. Fabrication of Conjugated Polymeric Nanoparticle

The NPs were conjugated with GPIbα (R & D Systems, Minneapolis, MN, USA) or anti-CD34 HuscFv with carbodiimide reaction by 1-ethyl-3-(3-dimethylaminopropyl) carbodiimide (EDC) and N-hydroxysulfosuccinimide (Sulfo-NHS). Briefly, lyophilized NPs were resuspended in 2-(4-morpholino)ethanesulfonic acid (MES) buffer, pH 4.75. Four hundred millimolars of EDC and 100 mM Sulfo-NHS were added to NP dispersion, then the mixture was kept at room temperature for 4 h. After that, excess EDC and NHS were removed using the supercentrifugation speed (30,000× *g*) at 4 °C for 30 min. The activated NP was washed with PBS. The NHS-activated NP pellet was resuspended with PBS and reacted with particular protein ligands. The mixture reaction was kept at 4 °C overnight. The unbound ligands were removed using supercentrifugation, then the supernatant was collected to measure the efficiency of the conjugation. The conjugated NP pellet was resuspended in PBS, and then immediately used for the experiments.

### 4.8. Nanoparticle Characterization

The fabricated NPs were resuspended in dH_2_O with the sonication and were characterized to determine the size and zeta potential (surface charge) by dynamic light scattering (DLS) with a zetasizer (Malvern, DKSH, Zurich, Switzerland). For morphology observation, the lyophilized PLGA was coated over double-sided adhesive tape attached to a cylinder specimen stub. Then, this specimen stub was sputter-coated with a thin layer of gold using sputter coater. Then, NP morphology was examined by field emission scanning electron microscope (FE-SEM) (JSM-7610F, JEOL, Tokyo, Japan) at an accelerating voltage of 5 kV.

The encapsulation efficiency (EE) of loaded NPs was measured using UV-VIS spectrophotometry by collecting the supernatant of NPs centrifugation at 30,000× *g* for 30 min. The EE was determined by C6 fluorescence intensity (excitation: 444 nm, emission: 510 nm) and RhB absorbance (520 nm). The percentage of EE was calculated using the following equation.

% Encapsulation efficiency = (initial feeded dye − free dye in supernatant)/(initial feeded dye) × 100(1)

The conjugation efficiency (CE) of conjugated NPs was determined from free remaining protein ligands in the supernatant using Bradford protein assays (Bio-Rad) according to the manufacturer’s instructions. Control NP sample served as sample blank. The percentage of CE was calculated using the following equation.

% Conjugation efficiency = (initial feeded ligand − free ligand in supernatant)/(initial feeded ligand) × 100(2)

### 4.9. Biocompatibility Study

HUVECs were established in wells of a 96-well culture plate (20,000 cells per well), and incubated at 37 °C, 5% CO_2_ overnight. Then, the cells were incubated with different NP samples at the particle concentrations of 0, 0.1, 0.25, 0.5, 1 mg/mL at 37 °C for 24 h. After incubation, cell viability was measured by CellTiter^®^ AQueous One (MTS) assay (Promega, Madison, WI, USA) according to the manufacturer’s instructions.

The hemocompatibility study was approved by the Ethics Committee of Human Research Protection Unit, Faculty of Medicine Siriraj Hospital, Mahidol University (the number of COA is Si 642/2018). The hemocompatibility of fabricated NPs was evaluated by blood clotting and hemolysis tests according to previously reported protocols [73]. In the blood clotting test, the anticoagulated blood was re-calcified with 0.1 M calcium chloride (Merck, Rahway, NJ, USA) to initiate the clotting reaction, and mixed immediately with NP suspension at the concentration of 0.5 and 1 mg/mL. The reaction was kept at room temperature for 10, 30, and 60 min. Thereafter, the unclotted red blood cells were lysed with dH_2_O and measured for the released hemoglobin at the absorbance 540 nm using a microplate reader to determine blood clotting. For the hemolysis test, the diluted anticoagulated blood was incubated with NP samples at 37 °C under gentle agitation for 2 h. Then, the blood samples were centrifuged at 1000× *g* for 10 min, and the absorbance of the supernatant was measured at 545 nm for hemolysis determination.

### 4.10. Binding of HuscFv-Conjugated NPs (HuscFv-NPs) to CD34-Positive Cells

HUVECs and KG-1a cells were tested for binding with HuscFv-NPs. RhB-loaded NPs and anti-CD34-conjugated NPs (anti-CD34-NP) served as negative and positive control NPs, respectively. After harvesting, the cells were washed with FACS buffer, then blocked with 1% BSA and kept on ice for 30 min. Next, each NP sample was added to the tube containing (1 × 10^5^ cells) cells and kept at 4 °C for 1 h. Then, all samples were subjected to flow cytometric analysis by detecting of RhB fluorescence at the excitation wavelength 546 nm and emission wavelength 568 nm.

### 4.11. Binding of GPIb-Conjugated NPs (GPIb-NPs) to vWF

Human vWF (1.5 μg) was coated on well surface of a 96-well plate at 4 °C overnight. After blocking with 1% BSA at 37 °C for 1 h, the coated surface was incubated with GPIb-NPs loaded with coumarin-6 at 37 °C under gentle agitation for 2 h. The control C6-loaded NPs and the untreated group served as the controls. The surface was gently washed with wash buffer several times to remove unbound NPs. Finally, fluorescence intensity of the loaded C6 inside the vWF-bound NPs was determined by using a microplate reader. In addition, for the competition test, free vWF solution was mixed with NP concentration at 0.5 mg/mL before incubation with vWF-coated surface.

### 4.12. Platelet Binding Prevention Test

Platelet-rich-plasma (PRP) was isolated from the buffy coat sample obtained from Siriraj Blood Bank, Siriraj Hospital, Bangkok. The buffy coat was centrifuged at 300× *g* at room temperature for 30 min. Then, the plasma containing platelets was collected. To prepare platelet-poor-plasma (PPP), the PRP was further centrifuged at 300× *g* at room temperature for 15 min, and then the PPP supernatant was collected. Five micrograms of human vWF was coated on the well surface of a 96-well plate surface at 4 °C for 48 h. After blocking with 1% BSA at 37 °C for 1 h, the coated surface was incubated with NP samples and PRP at the final concentration of NP 1 mg/mL and PRP 4.4 × 10^6^ platelets per well at 37 °C with gentle agitation for 1 h. The PRP and PPP only served as controls. The surface was gently washed with PBS several times to remove unbound platelets. The bound platelets were quantified by the lactate dehydrogenase (LDH) assay using CytoTox 96^®^ Non-Radioactive Cytotoxicity Assay (Promega, WI, USA) according to the manufacturer’s instructions.

### 4.13. Statistical Analysis

Statistical analysis was determined by one/two-way ANOVA using GraphPad Prism statistical program version 8 (GraphPad Software Inc., San Diego, CA, USA). Data were presented as mean ± standard deviation (SD). Differences were considered statistically significant at *p* < 0.05.

## 5. Conclusions

In this study, the novel human single-chain variable antibody fragment (HuscFv) recognizing CD34-expressing cells was produced successfully by the phage display technology. PLGA NPs conjugated dually with GPIb and HuscFv were fabricated successfully. The dually conjugated NPs were cyto-and hemo-compatible. The GPIb-NPs could target vWF and reduce platelet adherence to vWF. Furthermore, the HuscFv-NPs could capture CD34-positive cells, i.e., HUVEC and KG-1a cell lines, as the model of EPCs. Thus, the development of dual-NPs may provide a promising strategy for coating onto the vascular implant surfaces to reduce restenosis and simultaneously stimulate endothelium regeneration. In addition, the NPs may also be loaded with therapeutic agents, such as growth factors, to improve the rate of endothelium regeneration.

## Figures and Tables

**Table 1:** Sizes, polydispersity indices and zeta potentials of fabricated NPs by DLS.

NPs	Size (nm)	Polydispersity Index	Zeta Potential (mV)	%EE	%CE
Unloaded	192.3 ± 49.0	0.03 ± 0.01	−31.3 ± 7.2	-	-
C6-loaded	188.7 ± 49.6	0.04 ± 0.03	−39.4 ± 7.1	89.3 ± 0.3	-
RhB-loaded	198.9 ± 52.5	0.03 ± 0.03	−39.3 ± 8.0	48.8 ± 0.3	-
Control	199.7 ± 47.8	0.01 ± 0.01	−29.7 ± 9.6	-	-
GPIb	200.2 ± 50.6	0.02 ± 0.03	−40.8 ± 7.8	-	40.1 ± 24.3
HuscFv	202.3 ± 52.0	0.05 ± 0.02	−41.6 ± 6.9	-	38.2 ± 24.5
Dual ligands	216.6 ± 64.0	0.07 ± 0.01	−38.9 ± 7.8	-	61.3 ± 20.2

## Data Availability

The data presented in this study are available within the article.
